# Genetic diversity of human RNase 8

**DOI:** 10.1186/1471-2164-13-40

**Published:** 2012-01-24

**Authors:** Calvin C Chan, Jennifer M Moser, Kimberly D Dyer, Caroline M Percopo, Helene F Rosenberg

**Affiliations:** 1 Laboratory of Allergic Diseases, National Institute of Allergy and Infectious Diseases, National Institutes of Health, Bethesda, Maryland, USA; 2 Current address: Health Science Specialist, Genome Medicine Program, Department of Veterans Affairs, 810 Vermont Avenue, NW, Washington, D.C; 3 Building 10, Room 11C215, Laboratory of Allergic Diseases, NIAID, National Institutes of Health, 9000 Rockville Pike, Bethesda, Maryland 20892

**Keywords:** ribonuclease, polymorphism

## Abstract

**Background:**

Ribonuclease 8 is a member of the RNase A family of secretory ribonucleases; orthologs of this gene have been found only in primate genomes. RNase 8 is a divergent paralog of RNase 7, which is lysine-enriched, highly conserved, has prominent antimicrobial activity, and is expressed in both normal and diseased skin; in contrast, the physiologic function of RNase 8 remains uncertain. Here, we examine the genetic diversity of human RNase 8, a subject of significant interest given the existence of functional pseudogenes (coding sequences that are otherwise intact but with mutations in elements crucial for ribonucleolytic activity) in non-human primate genomes.

**Results:**

RNase 8 expression was detected in adult human lung, spleen and testis tissue by quantitative reverse-transcription PCR. Only two single-nucleotide polymorphisms and four unique alleles were identified within the RNase 8 coding sequence; nucleotide sequence diversity (π = 0.00122 ± 0.00009 per site) was unremarkable for a human nuclear gene. We isolated transcripts encoding RNase 8 via rapid amplification of cDNA ends (RACE) and RT-PCR which included a distal potential translational start site followed by sequence encoding an additional 30 amino acids that are conserved in the genomes of several higher primates. The distal translational start site is functional and promotes RNase 8 synthesis in transfected COS-7 cells.

**Conclusions:**

These results suggest that RNase 8 may diverge considerably from typical RNase A family ribonucleases and may likewise exhibit unique function. This finding prompts a reconsideration of what we have previously termed functional pseudogenes, as RNase 8 may be responding to constraints that promote significant functional divergence from the canonical structure and enzymatic activity characteristic of the RNase A family.

## Background

RNase A ribonucleases are vertebrate proteins with unique tertiary structure and specific enzymatic activity [1,2]. Bovine pancreatic ribonuclease, or RNase A, is the prototype and founding member of this family, which includes enzymatically-active proteins from humans, multiple mammalian species, birds, and vertebrate fish [3-5]. Initial publication of the complete sequence of the human genome led to the discovery of several highly divergent orthologs of RNase A (RNases 9-13) that seemed to have lost elements crucial for enzymatic activity [6,7]. Even among the more prototypical RNase A ribonucleases, such as eosinophil cationic protein (RNase 3) and avian leukocyte RNase A-2, ribonuclease activity is not an essential component of their prominent antimicrobial properties [8,9]. These and related findings have suggest that active evolutionary constraints may not be directed toward maintaining optimal levels of enzymatic activity, and that the RNase A genes themselves may actually be serving more as evolutionary scaffolds, the genetic raw material for ongoing diversification [9,10]. Examples of this concept include the functional compartmentalization of the digestive ribonucleases RNase 1 and 1B of herbivorous primate, douc langur [11]; the species-limited duplication of bovine RNase A leading to bovine seminal RNase [12]; the identification of multiple rodent RNase 1 clusters [13]; and the dramatic and extensive diversification of both primate and rodent eosinophil ribonucleases [14-16].

RNases 7 and 8 are among the more recently discovered of the RNase A ribonucleases. RNase 7 was described by Harder and Schröeder [17] as an antimicrobial protein from human skin and also by Zhang and colleagues [18] who located the RNase 7 open reading frame in the first draft release of the human genome sequence. RNase 7 is expressed in primary keratinocytes in response to proinflammatory stimuli [17], has dramatic activity against numerous bacterial and some fungal pathogens [17,19,20], and has been associated with several cutaneous disease states [21,22]. In contrast to the arginine-rich antimicrobial RNases ECP and leukocyte RNase A-2, RNase 7 is cationic due to an abundance of lysine residues. Huang and colleagues [23] have shown that multiple lysines interact with the bacterial membrane to promote antimicrobial activity; recently, *P. aeruginosa *outer membrane protein 1 has also been documented as a specific target [24]. RNase 7 coding sequences have been identified in recent releases of the chimpanzee (*P. troglodytes*) and macaque (*M. mulatta*) genomes, at 99% and 93% encoded amino acid sequence identities to the human RNase 7, respectively. There is no ortholog of RNase 7 in the mouse genome.

Human RNase 8 was also identified as an open reading frame in the first release of the human genome sequence [25]. In our original report, we noted that RNase 8 was not as cationic as RNase 7, and had an atypical cysteine structure later shown by Zhang [26] to have evolved by a gain-and-loss, or disulfide shuffling mechanism. We evaluated RNase 8 sequences from 10 primate species, and found several examples in which otherwise full length sequences encoded alterations in elements essential to ribonuclease structure and/or activity, a feature we termed "functional pseudogenes" [25]. This finding appears to be unique to RNase 8; no evidence of functional pseudogenization has emerged in similar studies of the eosinophil ribonucleases, angiogenin, or RNase 6 [14,27,28].

Given this unusual pattern of diversification, we questioned whether we might detect similar pseudogenized alleles of RNase 8 within the human population. We explored this possibility by isolating and sequencing coding alleles from characterized panels of genomic DNA from diverse human sources. Here we present our findings on the genetic diversity of human RNase 8. Equally interesting, we present a novel and previously unrecognized distal translational start site encoded within the transcript, which has prompted us to reconsider RNase 8 expression and function.

## Results

### Expression of RNase 8 in human tissues

**Figure 1:**
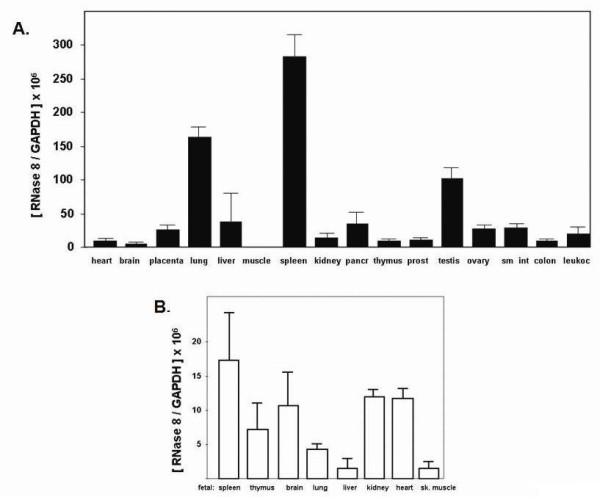
**Expression of RNase 8 in adult and fetal human tissues**. Copies of RNase 8 transcript per copy of GAPDH (x 10^6^) in (A) adult and (B) fetal tissues. Shown are the mean ± standard deviations of three separate experimental trials performed on pooled cDNAs. Abbreviations include pancr, pancreas; prost, prostate; sm int, small intestine; leukoc, leukocyte; sk muscle, skeletal muscle.

Expression of RNase 8 in adult and fetal human tissue was evaluated by qRT-PCR Figure 1A and 1B using standard curves for quantitative determination of absolute copy number of both RNase 8 and the expression control, GAPDH (see Methods). As shown, expression of RNase 8 (copies/GAPDH) was relatively low in all tissues evaluated. Even at its most abundant, in adult human spleen, we detected 1 copy of RNase 8 to every 4000 copies of GAPDH; expression in other cells and tissues was substantially lower. Interestingly, our previous examination of RNase 8 by Northern analysis suggested that prominent expression was limited to placental tissue. The reasons underlying the discrepancy between these two sets of results remains unclear, but are likely related to methodology. Quantitative RT-PCR is a more sensitive and specific means for detection of gene expression. The method featured here has undergone careful development and cross-reaction specifically with human RNase 7 has been ruled out (data not shown). As the target tissues are from commercial suppliers, and the Northern blot featured in the earlier publication was utilized for studies carried out nearly ten years ago [25], it is not possible to do a direct comparison at this time.

### The coding sequence of human RNase 8 includes two SNPs and an extended amino terminus

**Figure 2:**
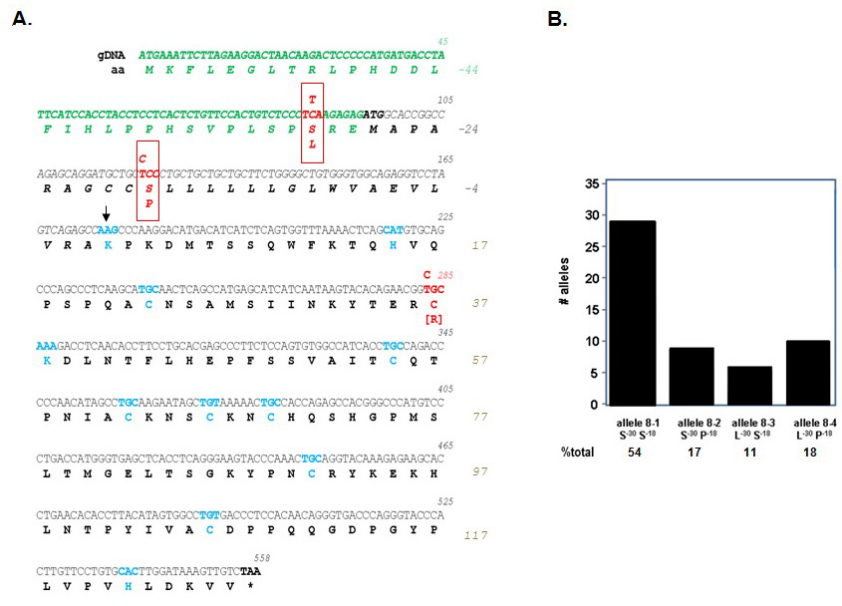
**The coding sequence of human RNase 8 includes two non-silent SNPs and a previously unrecognized 5' extended open reading frame**. **(A) **Highlighted in red are SNPs located within the hydrophobic segment previously identified as a signal sequence (in black, in italics) another within the newly-identified coding segment preceding it (in green, in italics). Highlighted in blue are the amino terminus (K) of the RNase domain (at the arrow), the eight cysteines and catalytic histidines (H) and lysine (K); stop codon denoted by asterisk (*) and bold text. **(B) **Number of independent alleles and percent of total are as shown. GenBank accession numbers for sequences features in this figure are JQ361124-JQ361128.

A 462 bp open reading frame including a hydrophobic sequence (here, nt 94-174) preceding the ribonuclease domain (nt 115-555) was originally identified in the human genome as RNase 8 based on its direct sequence homology with human RNase 7 [25]. In this work, we re-examined this locus, and we identified an extended open reading frame which includes a distal translational start site and a segment encoding an additional 30 amino acids preceding the original amino terminus Figure 2A. This extended 555 bp open reading frame was identified in sequences isolated from 27 independent samples of human genomic DNA, providing 54 unique alleles for analysis. All alleles encoded complete open reading frames without frameshift mutations or premature stop codons. No changes were detected in elements necessary for enzymatic activity (the H^22 ^- K^45 ^- H^129 ^catalytic triad), although one allele from one individual included a polymorphism/mutation that resulted in a C^37 ^/R (codon T^283 ^↔ C). This would be a structurally-destabilizing mutation in a typical RNase A family ribonuclease; however, given unusual amino terminus encoded by the complete open reading frame within the RNase 8 transcript (see below), it is not at all clear what the impact of this polymorphism might be on its final conformation.

Two non-silent SNPs were identified within the extended RNase 8 coding sequence. One SNP (C^86 ^↔ T; rs12434982) results in alternating S^-30^/L within the extended amino terminal sequence; the second SNP (T^121 ^↔ C) alters the sequence of the hydrophobic segment, S^-18 ^/P (rs12437266; [29]). Other than the aforementioned, previously unreported single allele (C^37^/R), there were no SNPs identified in the ribonuclease domain of RNase 8. The predominant S^-30^S^-18 ^variant accounts for 54% of the alleles examined; the remaining variants constitute 17% (S^-30^P^-18^), 11% (L^-30^S^-18^), and 18% (L^-30^P^-18^) of the total Figure 2B. Nucleotide sequence diversity was calculated at π = 0.00122 ± 0.00009 per site.

### Characterization of the transcripts encoding human RNase 8

**Figure 3:**
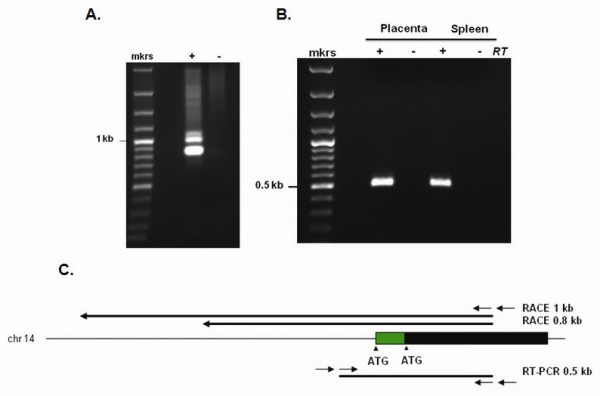
**Identification and characterization of RNase 8 transcript**. **(A) ~**800 and ~1000 bp transcripts encoding RNase 8 were isolated from human spleen cDNA using a nested rapid amplification of cDNA ends (RACE); +, including adapter primer;-without adapter primer. **(B) **Findings from RACE confirmed by RT-PCR using two unique sources of poly A^+ ^RNA; +, including reverse transcriptase;-without reverse transcriptase. **(C) **RNase 8 on human chromosome 14; black bar, coding sequence originally described [25] green bar, novel amino terminal extension. RACE amplification products are shown above extending 0.8 and 1 kb from primer within the RNase 8 coding sequence; nested RT-PCR amplification product (0.5 kb) is also as shown below. The GenBank accession number for the spleen cDNA RNase 8 transcript sequences is JQ353679.

Transcripts encoding RNase 8 were detected in and amplified from human spleen cDNA using the rapid amplification of cDNA ends (RACE) method Figure 3A. The ~800 bp and ~1000 bp sequences amplified the full coding sequence of RNase 8, including sequence encoding the 5' amino terminal sequence including the distal start site; no introns were detected. To confirm the presence of the amino terminal extension and to rule out the possibility of genomic contaminants in the commercial cDNA preparation, we created new 5'→ 3' primers based on cDNA sequence determined from RACE together with known 3'→ 5' primers Table 1 to carry out nested RT-PCR targeting both human placenta and spleen poly A^+ ^RNA Figures 3B and 3C. This experimental trial (performed with and without reverse transcriptase) documented expression of RNase 8 transcript in these tissues and likewise amplified the full open reading frame including the amino terminal extension Figure 3C.

**Table 1:** Amplification, sequencing and site-directed mutagenesis primers for RNase 8

**Gene-amplification**	
RN8F	5'- CAC ATT GCC CTG CAA TAA CTG GCT -3'
RN8R	5'- AGG TTG AGT GTG TGG GAG GGA AAT -3'
**Sequencing**	
RNase 8 Fi	5'- TCA CTC TGT TCC ACT GTC TCC CTT -3'
RNase 8 Ri	5'- AGT CTG CAG AGT GTG AG GTG GAA -3'
**Human RNase 8 nested RACE (human spleen cDNA)**	
rcRN8 (3' → 5')	5'-CAA CTT TAT CCA AGT GCA CAG GA-3'
R85Rn (3' → 5')	5'-CTG GGT CAC CCT GTT GTG GAG-3'
**Human RNase 8 nested reverse-trancriptase PCR (RT-PCR)**	
R8S	5'-ACA ATA GAA TGC CAG GGG TGT TCA-3'
R85R	5'-CAA CTT TAT CCA AGT GCA CAG GA-3'
R8Sn	5'-CAT TGC CCT GCA ATA ACT GGC TT-3'
R85Rn	5'-CTG GGT CAC CCT GTT GTG GAG-3'
**Human RNase 8 quantitative RT-PCR**	
RNase 8fx	5'- CAT AGC CTG CAA GAA TAG CTG TAA AA-3'
RNase 8rx	5'- TCA CCC ATG GTC AGG GAC AT-3'
6FAM-RNase 8-TAMRA	6FAM-CTG CCA CCA GAG CCA CGG GC-TAMRA
**Nested primate genome RNase 8**	
5'R8F	5'-GCC AGG GGT GTT CAA TAT CTT AG-3'
3'R8R	5'-AGG TTG AGT GTG TGG GAG GGA AAT-3'
5'R8Fn	5'-CAC ATT GCC CTG CAA TAA CTG GCT-3'
3'R8Fn	5'-AGT CTG CAG AGT GTG AGG TGG AAC-3'
**RNase 8 expression plasmids**	
**CCC3**	
R8D	5'-AAG CTT ATT GCC CTG CAA TAA CTG GCT TAG GGT-3'
R8C	5'-CTC CTC CTC TCT AGA GAC AAC TTT ATC CAA GTG CAC A-3'
**CCC4**	
R8O	5'-AAG CTT ACT CTG TTC CAC TGT CTC CCT TAA GAG AG-3'
R8C	5'-CTC CTC CTC TCT AGA GAC AAC TTT ATC CAA GTG CAC A-3'
**CCC5 mutagenesis**	
R85F	5'-TCT CCC TTA AGA GAG ATA GCA CCG GCC AGA GCA-3'
R85R	5'-TGC TCT GGC CGG TGC TAT CTC TCT TAA GGG AGA-3'

### Extended amino termini in primate RNase 8s

**Figure 4:**
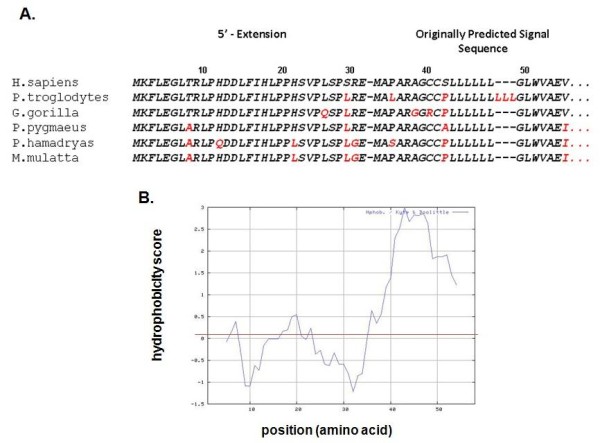
**Predicted amino terminal extensions of the RNase 8 coding sequence are conserved in primate genomes**. **(A) **Shown are coding sequences that incorporate RNase 8 from Build 2.1 of the genome of *P. troglodytes *and Build 1.1 of *M. mulatta *and original sequence data generated from RNase 8 isolated from genomic DNA from *P. troglodytes *(chimpanzee; JQ353681), *P. pygmaeus *(orangutan; JQ353682), *P. hamadryas *(babooon' JQ353680), and *G. gorilla *(gorilla; JQ353683). Amino acids 1-31 are from the extended open reading frame, amino acids 32-55 (separated by hyphen) are those originally predicted as components of a signal sequence; divergence from the *H. sapiens *(human) sequence is indicated in red. (**B) **Evaluation of the predicted hydrophobicity of the human RNase 8 amino terminus (amino acids 1-55) via the Kyte-Doolittle algorithm [29].

Extended amino terminal sequences were identified in the open reading frames of RNase 8s from five higher primate species Figure 4A. Hydrophobicity score vs. amino acid position (Kyte-Doolittle plot [30], Figure 4B) indicates that this segment (amino acids 1-31, note the renumbering in this figure) is relatively hydrophilic, particularly in comparison to the hydrophobic segment that follows (amino acids 32-55), although the multiple leucine substitutions in the *P. hamadryas *and *M. mulatta *sequences render this region somewhat less hydrophilic than the featured human sequence. Overall, however, the presence of this amino terminal sequence indicates that the encoded RNase 8 polypeptide may be structurally atypical among RNase A family members, as this configuration is not one of a classic secretory mediator (see Discussion).

### Characterization of the two translational start sites

**Figure 5:**
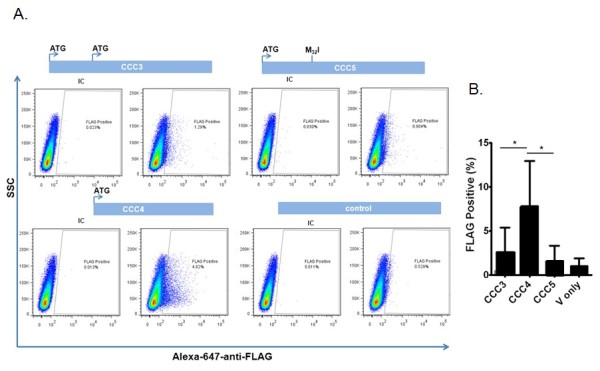
**Evaluation of the distal and proximal translational start sites**. **(A) **COS-7 cells were transfected via the lipofectamine method, and cells expressing carboxy-terminal-FLAG tagged RNase 8 were detected by flow cytometry. Construct CCC3 includes both distal and proximal start sites (see Figure 3); constructs CCC4 and CCC5 include the proximal and distal translational start sites only, respectively, and the final panel is the vector (pcDNA3.1) control alone. IC; rabbit anti-IgG isotype control. **(B) **Compilation of seven independent experiments; *p < 0.05.

As shown in Figure 2, the full-length RNase 8 transcript encodes both proximal (at amino acid position -27) and distal (at position -58) translational start sites. The proximal is in a position that is typical for RNase A family ribonucleases, as it leads what would otherwise be a hydrophobic signal sequence; its nucleotide sequence is within a reasonable approximation of a consensus translation initiation sequence. In contrast, the distal translational start site is atypical and requires functional assessment. Toward this end, we utilized one or both translational start sites to direct synthesis of recombinant RNase 8 in COS-7 cells Figure 5. Quantitative RT-PCR analysis indicated that all mRNAs were transcribed and stable in COS-7 cells, with C_t _values between 20 and 23 (data not shown). As anticipated, the RNase 8 proximal translational start site viable and active (construct CCC4). The distal start site alone (construct CCC5) was likewise active. Interestingly, the construct that directly replicates the configuration of the natural transcript (CCC3), and includes both the distal and the proximal translational start sites, promotes expression of RNase 8 protein over background, albeit at a reduced level.

## Discussion

RNase 8 is closely related to RNase 7, but it is clearly subject to different evolutionary constraints. RNase 7 is a prototypical RNase A ribonuclease, as it is enzymatically active against standard RNA substrates, includes an H -K- H catalytic triad, and eight canonically-spaced cysteines that generate disulfide bonds that define the tertiary structure [17,18]. Although no formal evolutionary analysis has been performed on the RNase 7 lineage, a comparison of available coding sequences from human and genome builds of chimpanzee and macaque [29] suggests that this lineage is highly conserved over time. In contrast, in an earlier study of RNase 8, we documented substantial interspecies divergence (1.3 × 10^-9 ^substitutions/site/year), and we noted the existence of true pseudogenes, with significant truncations in encoded polypeptides in the genomes of several higher primates, including gorilla (*G. gorilla*) and baboon (*P. hamadryas*). We also identified RNase 8 functional pseudogenes; these were genes that encoded full length RNase 8 polypeptides but with mutations in crucial structural or catalytic residues [25]. Examples include RNase 8s from the tamarin (*S. oedipus*), in which the first catalytic histidine (H) is mutated to aspartic acid (D), and likewise the African green monkey (*C. aethiops*), in which a structural cysteine is lost. We embarked on the present study in large part to determine whether alleles encoding pseudogenes (true or functional) might be circulating within the human population. Interestingly, although detected in only one human allele among those in our study, we did identify a C^37^/R polymorphism similar to that found in the *C. aethiops *gene. Aside from that one isolate, only two main SNPs were identified within the coding sequence of RNase 8, and although the nucleotide sequence diversity was somewhat greater than that observed for RNase 7 (π = 0.00073 ± 0.00022; unpublished data), the value calculated for RNase 8, π = 0.00122 ± 0.00009 per site, is within the normal range for nuclear genes [31], and is similar to values determined for the functional eosinophils RNases, the eosinophil-derived neurotoxin (EDN/RNase 2; π = 0.00063 ± 0.0001) and eosinophil cationic protein (ECP/RNase 3; π = 0.00113 ± 0.00006) [32].

However, perhaps most intriguing of all is the elucidation of the distal translational start site and putative extended open reading frame of RNase 8; the original start site was identified based on sequence homology to RNase 7 [25] and other RNase A family ribonucleases. This amino terminal extension is unique and breaks the paradigm for the prototypical RNase A ribonucleases, which, as a group, are granule and/or secretory proteins, with amino terminal hydrophobic signal sequences that direct the nascent polypeptides through the ER/Golgi for post-translational processing prior to release [33]. In contrast to the hydrophobic quality of amino acids 32 to 58 Figure 5B, the amino terminus that includes amino acids 1-31, has a predominantly hydrophilic nature. As such, RNase 8 with this extension may not be a secretory protein. Unfortunately, despite significant effort and a recently developed specific anti-peptide antibody, we have been unable to detect expression of RNase 8 polypeptide in human fibroblast or hematopoietic cell lines. This may be due to the fact that expression levels at homeostasis are quite low Figure 2 and the appropriate conditions under which expression is augmented are not yet clear. Consistent with this finding, we have found that the natural construct, which includes the distal translational start site in tandem with the proximal translational start site, promotes only minimally activity Figure 5. The absence of an RNase 8 ortholog in laboratory rodent models adds to the limitations we are confronting.

## Conclusion

Given these observations, taken together with our recent appreciation of the RNase A ribonuclease genes as scaffolds for evolutionary change [9,10], it is apparent that we need to consider the possibility that RNase 8 is expressed as something other than a standard secretory ribonuclease; by extension, we need to revise the concept of functional pseudogenes. It is conceivable that RNase 8 is under constraints promoting selection of a novel function, perhaps only partially-related to its original role; as such, rigid adherence to the structure that is required for enzymatic cleavage of polymeric RNA is no longer necessary or useful. This and related hypotheses certainly merit further consideration.

## Methods

### Human genomic DNA samples and sequence analysis

Genomic DNA samples from human lymphocytes were obtained from the Coriell Institute for Medical Research (Camden, NJ). Additional samples of genomic DNA were obtained from normal volunteers after standard informed consent (protocol NIAID 09-I-0049). Table 1 includes sequences of all amplification, sequencing and mutagenesis primers. Briefly, 50 ng genomic DNA was used as an amplification target with 1 unit of Platinum Taq polymerase under standard conditions. Genomic DNAs, Taq proofreading polymerase, and gene-specific primers RN8F and RN8R primers were used to amplify a 688 bp genomic fragment encompassing the RNase 8 open reading frame. Amplification products were sequenced in both directions with BigDye terminator v3.1 on a 3130x Applied BioSystems sequencer using manufacturer's specifications. Heterozygosity was confirmed and resolved as necessary by cloning amplification products into TA vectors and re-sequencing.

### Amplification of RNase 8 from primate genomic DNA

Primate genomic DNAs from *Pan troglodytes, Gorilla gorilla, Pongo pygmaeus *were obtained from BIOSLabs (New Haven, CT). Genomic DNA from *Papio hamadryas *was isolated from the 26CB-1 lymphoblast line (CRL-1495, American Type Culture Collection, Manassas, VA). Nested PCR was carried out via first round amplification using primers 5'-R8F and 3'-R8R; 0.5 μg of gDNA and 50X Titanium Taq polymerase were used to amplify the target gene with the cycling parameters including 95°C for 1 min followed by 30 cycles, 95°C for 30 sec, then 68°C 1.5 min, and 68°C for 3 min. The second nested amplification was performed to generate a ~ 625 bp product, also with Titanium Taq polymerase and primers 5'-R8Fn and 3'-R8Rn. Amplification products were cloned into TA 2.1 vector (Invitrogen) and multiple clones were sequenced in both directions using BigDye terminator v3.1 on a 3130x Applied Biosystems sequencer.

### Database search and analysis of genome sequence data

Primate RNase 7 and RNase 8 sequences are located in the GenBank database under accession numbers AF473854-AF473863. Extended 5' coding sequences were identified in RNase 8s of both *P. troglodytes *and *M. mulatta *upon BLAST search of these full genome databases (Builds 2.1 and 1.1, respectively) accessed via the National Center for Bioinformatics (NCBI) website [29]. Sequence alignments were generated with ClustalW [34] and analyzed with algorithms included within MEGA version 4.0 ([35] and DNAsp [36].

### Rapid amplification of cDNA ends (RACE)

0.5 ng of human spleen marathon RACE-ready cDNA (Clontech) was amplified using the A2 polymerase kit (Clontech) and a 3' → 5' primer rcRN8 specific for RNase 8 and primary adapter primer as per manufacturer's instructions. The PCR cycling parameters were as follows: 94°C for 1 min followed by 40 cycles of 94°C for 30 sec and then 62°C for 2 min. 1 μL of amplified product was then used in a nested 5'RACE reaction targeting RNase8 using the nested 3' → 5' R85Rn gene-specific primer and nested adapter primer. The following cycling parameters were used for the nested RACE: 94°C for 1 min followed by 35 cycles of 94°C for 30 sec. and then 62°C for 2 min. This method yielded two amplification products, ~0.8 kb and ~1 kb, which were both cloned into TA 2.1 vectors; multiple clones were sequenced in both directions using BigDye terminator v3.1 on a 3130x Applied Biosystems sequencer according to manufacturer's instructions.

### Reverse transcriptase polymerase chain reaction (RT-PCR) detection of RNase 8

Human spleen and placenta poly A^+ ^RNA (Clontech) were used to generate cDNA with a 1^st ^strand cDNA synthesis kit (Roche) following manufacturer's instructions. Briefly, 1 μg of RNA was DNase I-treated (Invitrogen) according to manufacturer's instructions; 100 ng of DNase-treated poly A^+ ^RNA was reverse transcribed into cDNA using the following parameters: 25°C for 10 min, then 42°C for 60 min followed by 99°C for 5 min., and finally 4°C for 5 min. Control (no reverse transcriptase (RT)) samples were also generated. PCR amplification of RNase8S fragment was performed using cDNA from human spleen or placenta (or no RT controls), Titanium Taq polymerase kit, and primers R8S and R85R with the following cycling parameters: 95°C for 1 min, then 30 cycles of 95°C for 30 sec followed by 68°C 1.5 min., and finally 68°C for 3 min. From the RNase8S amplification product, nested PCR was performed using Titanium Taq polymerase and primers R8Sn and R85Rn which generated a ~544 bp fragment with the following cycling parameters 95°C for 1 min, 30 cycles, 95°C for 30 sec, 68°C 1.5 min, and 68°C for 3 min. The amplification products were cloned into TA 2.1 vector and multiple clones were sequenced in both directions using BigDye terminator v3.1 on a 3130x Applied Biosystems sequencer according to manufacturer's instructions.

### Quantitative reverse-transcription polymerase chain reaction (qPCR) detection of RNase 8 expression in human tissue

Human and fetal tissue cDNA panels (Clontech) were screened using TaqMan universal PCR master mix and ABI 7500 Real Time PCR system according to manufacturer's instructions. The following primer/probe set was used to detect RNase 8,

primers, RNase 8fx, RNase 8rx; probe, 6FAM-RNase 8-TAMRA. The primer/probe set was specific for RNase 8, and did not detect RNase 7 plasmid DNA (data not shown). Each of the aforementioned tissue samples was evaluated in triplicate along with a single no-template control. qPCR screening by this method was also performed on human spleen and placenta cDNA generated from poly A^+ ^RNA following the same procedure. Absolute quantities were determined by interpolation to a standard curve of 10 to 10^10 ^copies RNase 8 coding sequence in the pCR 2.1 vector (Invitrogen), run in duplicate. Human GAPDH was detected via standard primer/probe set (Applied Biosystems) with absolute quantities determined by interpolation to a standard curve (nucleotides 41-1425 of the human GAPDH coding sequence, GenBank # NM_002046 cloned into pCR 2.1) run in duplicate.

### RNase 8 expression plasmids, transfection and intracellular staining-flow cytometric analysis

RNase 8 expression constructs CCC3, CCC4 and CCC5 were prepared. CCC3 is a full length 595 bp RNase 8 open reading frame with both distal and proximal translational start sites and a carboxy-terminal FLAG (Figure 2). CCC4 is shortened 550 bp open reading frame that includes only the proximal translational start site. CCC5 is identical to CCC3 save for a point mutation that converts the methionine (ATG) in the proximal translational start site to an isoleucine. These open reading frames were cloned into the Hind III and Xho I restriction sites of expression plasmid pcDNA3.1 (Invitrogen) and purified (Origene) for transfection into COS-7 cells. COS-7 cells (ATCC CRL-1651) were seeded at a density of 3 × 10^6 ^cells per well in a six well plate 24 hours prior to transfection. 1 μg of DNA mixed with Lipofectamine 2000 reagent (Invitrogen) in a 1:3 ratio was added dropwise to each well. Six hours after addition of the transfection mixture, fresh media was added. 48 hours post-transfection, cells were removed from the plate with 0.05% Trypsin (Invitrogen) and 2 × 10^5 ^cells were added to a polystyrene tube (Falcon), fixed with 4% paraformaldehyde (Thermo Scientific), and washed 3 times with ice-cold 0.1% PBS/BSA. Cells were then washed 3 times with 0.1% PBS/Saponin solution and blocked with 5% milk in 0.1% PBS/saponin for 30 minutes on ice. After blocking, cells were stained with either rabbit anti-FLAG antibody conjugated to Alexa 647 (Cell Signaling) or anti-rabbit conjugated to Alexa 647 (Cell Signaling), both diluted 1:100 in 0.1% PBS/saponin for 30 minutes on ice. Cells were then washed 3 times in PBS/BSA 0.1% and one hundred thousand events were collected using an LSRII flow cytometer (BD Biosciences) and data collected was analyzed by FlowJo (Tree Star).

## List of Abbreviations

**RNase**: ribonuclease; **SNP**: single-nucleotide polymorphism; **RT-PCR**: reverse-transcriptase polymerase chain reaction; **RACE**: rapid amplification of cDNA ends; **ECP**: eosinophil cationic protein; **EDN**: eosinophil-derived neurotoxin; **nt**: nucleotide; **bp**: base pair.

## Authors' contributions

**CCC **contributed the experimental data in Figures 2-5 and reviewed and edited the manuscript, **JMM **contributed the experimental data in Figure 1 and reviewed and edited the manuscript, **KDD **provided ongoing supervision and technical oversight and reviewed and edited the manuscript, **CMP **finalized and confirmed experimental data and reviewed and edited the manuscript, and **HFR **identified the distal translational start site from data provided by JMM, created Figures and wrote the first draft of the manuscript. All authors read and approved the final version of the manuscript.

## Author information

The Rosenberg laboratory has a longstanding interest in the evolution and function of secretory ribonucleases, most notably of the RNase A family.

## References

[B1] Beintema JJ Introduction: the ribonuclease A superfamily Cell Mol Life Sci 1998 54 763 765 10.1007/s000180050204 9760984 PMC11147233

[B2] Dyer KD Rosenberg HF (2006) The RNase A superfamily: generation of diversity and innate host defense Mol Diversity 2006 10 585 597 10.1007/s11030-006-9028-2 16969722

[B3] Rosenberg HF RNase A ribonucleases and host defense: an evolving story J Leukoc Biol 2008 83 1079 1087 10.1189/jlb.1107725 18211964 PMC2692241

[B4] Cho S Zhang J Zebrafish ribonucleases are bactericidal: implications for the origin of the vertebrate RNase A superfamily Mol Biol Evol 2007 24 1259 1268 10.1093/molbev/msm047 17347156

[B5] Pizzo E Varcamonti M Di Maro A Zanfardino A Giancola C D'Alessio G Ribonucleases with angiogenic and bactericidal activities from the Atlantic salmon FEBS J 2008 275 1283 1295 10.1111/j.1742-4658.2008.06289.x 18279393

[B6] Penttinen J Pujianto DA Sipila P Huhtaniemi I Poutanen M Discovery *in silico *and characterization in vitro of novel genes exclusively expressed in the mouse epididymis Mol Endocrinol 2003 17 2138 2151 10.1210/me.2003-0008 12920233

[B7] Cho S Beintema JJ Zhang J The ribonuclease A superfamily of mammals and birds: identifying new members and tracing evolutionary histories Genomics 2005 85 208 220 10.1016/j.ygeno.2004.10.008 15676279

[B8] Rosenberg HF Recombinant human eosinophil cationic protein. Ribonuclease activity is not essential for cytotoxicity J Biol Chem 1995 270 7876 7881 7713881 10.1074/jbc.270.14.7876

[B9] Nitto T Dyer KD Czapiga M Rosenberg HF Evolution and function of leukocyte RNase A ribonucleases of the avian species, *Gallus gallus* J Biol Chem 2006 281 25622 25634 10.1074/jbc.M604313200 16803891

[B10] Pizzo E D'Alessio G The success of the RNase scaffold in the advance of biosciences and in evolution Gene 2007 406 8 12 10.1016/j.gene.2007.05.006 17616268

[B11] Zhang J Zhang YP Rosenberg HF Adaptive evolution of a duplicated pancreatic ribonuclease gene in a leaf-eating monkey Nature Genetics 2002 30 411 415 10.1038/ng852 11925567

[B12] D'Alessio G Di Donato A Parente A Piccoli R Seminal RNase: a unique member of the ribonuclease superfamily Trends Biochem Sci 1991 16 104 106 2057997 10.1016/0968-0004(91)90042-t

[B13] Dubois JY Catzeflis FM Beintema JJ The phylogenetic position of *Acomyinae *as sister group of *Murinae *and *Gerbillinae *clade: evidence from the nuclear ribonuclease gene Mol Phylogenet Evol 1999 13 181 192 10.1006/mpev.1999.0674 10508551

[B14] Rosenberg HF Dyer KD Tiffany HL Gonzalez M Rapid evolution of a unique family of primate ribonuclease genes Nature Genetics 1995 10 219 223 10.1038/ng0695-219 7663519

[B15] Zhang J Rosenberg HF Nei M Positive Darwinian selection after gene duplication in primate ribonuclease genes Proc Natl Acad Sci USA 1998 95 3708 3713 10.1073/pnas.95.7.3708 9520431 PMC19901

[B16] Zhang J Dyer KD Rosenberg HF Evolution of the rodent eosinophil-associated RNase gene family by rapid gene sorting and positive selection Proc Natl Acad Sci (USA) 2000 97 4701 4706 10.1073/pnas.080071397 PMC18296 10758160

[B17] Harder J Schröder JM RNase 7, a novel innate immune defense antimicrobial protein of healthy human skin J Biol Chem 2002 277 46779 467784 10.1074/jbc.M207587200 12244054

[B18] Zhang J Dyer KD Rosenberg HF Human RNase 7: a new cationic ribonuclease of the RNase A superfamily Nucl Acids Res 2003 31 602 607 10.1093/nar/gkg157 12527768 PMC140521

[B19] Harder J Schröder JM Antimicrobial peptides in human skin Chem Immunol Allergy 2005 86 22 41 15976486 10.1159/000086650

[B20] Koten B Simanski M Glaser R Podschun R Schröder JM Harder J RNase 7 contributes to the cutaneous defense against *Enterococcus faecium* PloS One 2009 4 e6424 10.1371/journal.pone.0006424 19641608 PMC2712763

[B21] Zanger P Holzer J Schleucher R Steffen H Schittek B Gabrysch S Constitutive expression of the antimicrobial peptide RNase 7 is associated with *Staphylococcus aureus *infection of the skin J Infect Dis 2009 200 1907 1915 10.1086/648408 19919305

[B22] Gambichler T Skrygan M Tomi H Othlinghaus N Brockmeyer NH Altmeyer P Kreuter A Differential mRNA expression of antimicrobial peptides and proteins in atopic dermatitis as compared to psoriasis vulgaris and healthy skin Int Arch Allergy Immunol 2008 147 17 24 10.1159/000128582 18446049

[B23] Huang YC Lin YM Chang TW Wu SJ Lee YS Chang MD Chen C Wu SH Liao YD The flexible and clustered lysine residues of human ribonuclease 7 are critical for membrane permeability and antimicrobial activity J Biol Chem 2007 282 4626 4633 17150966 10.1074/jbc.M607321200

[B24] Lin YM Wu SJ Chang TW Wang CF Suen CS Hwang MJ Chang MD Chen YT Liao YD Outer membrane protein I of *Pseudomonas aeruginosa *is a target of cationic antimicrobial peptide/protein J Biol Chem 2010 285 8985 8994 10.1074/jbc.M109.078725 20100832 PMC2838320

[B25] Zhang J Dyer KD Rosenberg HF RNase 8, a novel RNase A superfamily ribonuclease expressed uniquely in placenta Nucl Acids Res 2002 30 1169 1175 10.1093/nar/30.5.1169 11861908 PMC101240

[B26] Zhang J Disulfide-bond reshuffling in the evolution of an ape placental ribonuclease Mol Biol Evol 2007 24 505 512 17116644 10.1093/molbev/msl177

[B27] Zhang J Rosenberg HF Diversifying selection of the tumor-growth promoter angiogenin in primate evolution Mol Biol Evol 2002 19 438 445 10.1093/oxfordjournals.molbev.a004099 11919285

[B28] Deming MS Dyer KD Bankier AT Piper MB Dear PH Rosenberg HF Ribonuclease k6: chromosomal mapping and divergent rates of evolution within the RNase A gene superfamily Genome Res 1998 8 599 607 9647635 10.1101/gr.8.6.599

[B29] National Center for Biotechnology Information (NCBI) http://www.ncbi.nlm.nih.gov/

[B30] ExPasy/Kyte-Doolittle http://www.expasy.ch/cgi-bin/protscale.pl

[B31] Li W-H Sadler LA Low nucleotide diversity in man Genetics 1991 129 519 523 10.1093/genetics/129.2.513 PMC1204640 1743489

[B32] Zhang J Rosenberg HF Sequence variation at two eosinophil-associated ribonuclease loci in humans Genetics 2000 156 1949 1958 11102386 10.1093/genetics/156.4.1949 PMC1461363

[B33] MacDonald RJ Stary SJ Swift GH (1982) Rat pancreatic ribonuclease messenger RNA: the nucleotide sequence of the entire mRNA and the derived amino acid sequence of the pre-enzyme J Biol Chem 1982 257 14582 14585 7174650

[B34] ClustalW http://clustalw.genome.jp

[B35] Tamura K Dudley J Nei M Kumar S MEGA4: Molecular Evolutionary Genetics Analysis (MEGA) software version 4.0 Mol Biol Evol 2007 24 1596 1599 http://www.megasoftware.net 10.1093/molbev/msm092 17488738

[B36] DNAsp http://www.ub.es/dnasp

